# Depression Assessment Method: An EEG Emotion Recognition Framework Based on Spatiotemporal Neural Network

**DOI:** 10.3389/fpsyt.2021.837149

**Published:** 2022-03-15

**Authors:** Hongli Chang, Yuan Zong, Wenming Zheng, Chuangao Tang, Jie Zhu, Xuejun Li

**Affiliations:** ^1^Key Laboratory of Child Development and Learning Science, Ministry of Education, Southeast University, Nanjing, China; ^2^School of Information Science and Engineering, Southeast University, Nanjing, China

**Keywords:** depression, emotion recognition, electroencephalogram (EEG), convolutional neural network (CNN), long-short term memory network (LSTM)

## Abstract

The main characteristic of depression is emotional dysfunction, manifested by increased levels of negative emotions and decreased levels of positive emotions. Therefore, accurate emotion recognition is an effective way to assess depression. Among the various signals used for emotion recognition, electroencephalogram (EEG) signal has attracted widespread attention due to its multiple advantages, such as rich spatiotemporal information in multi-channel EEG signals. First, we use filtering and Euclidean alignment for data preprocessing. In the feature extraction, we use short-time Fourier transform and Hilbert–Huang transform to extract time-frequency features, and convolutional neural networks to extract spatial features. Finally, bi-directional long short-term memory explored the timing relationship. Before performing the convolution operation, according to the unique topology of the EEG channel, the EEG features are converted into 3D tensors. This study has achieved good results on two emotion databases: SEED and Emotional BCI of 2020 WORLD ROBOT COMPETITION. We applied this method to the recognition of depression based on EEG and achieved a recognition rate of more than 70% under the five-fold cross-validation. In addition, the subject-independent protocol on SEED data has achieved a state-of-the-art recognition rate, which exceeds the existing research methods. We propose a novel EEG emotion recognition framework for depression detection, which provides a robust algorithm for real-time clinical depression detection based on EEG.

## 1. Introduction

The recognition of emotion is a major research direction of affective computing, which had been widely used to detect depression (1, 2). Emotion is crucial to the quality and scope of human daily experience (3). With the development of the brain–computer interface (BCI) and the advancement of artificial intelligence, the recognition of emotions based on EEG signals has become an active research topic of emotion recognition. EEG signals contain a large amount of information related to emotions and have the characteristics of high time resolution, and are not effortless to disguise (4–6), which shows tremendous advantages in the field of real-time emotion recognition. Accurate and real-time judgment of human emotional state through some technical means has great application value in many areas, for example, driving fatigue detection (7), depression monitoring (8), and real-time monitoring of critically ill patients (9).

The relationship between EEG and emotion has been reported in past studies. Brain regions implicated in emotional experience include the orbitofrontal cortex, insular cortex, and anterior and posterior cingulate cortices. The amygdala is involved in linking perception with automatic emotional responses and memory (3). The activation of the amygdala seemed to be more related to negative emotions, and the relative activation of the right frontal lobe correlated with negative emotions (such as fear or disgust) (10). Precisely, fear corresponds to the amygdala (11), anger is related to the orbitofrontal cortex and anterior cingulate cortex (12), sadness occurs in the amygdala and right temporal pole (13), and disgust is produced in the anterior insula and anterior cingulate cortex (14). In addition, the power of the alpha band and the asymmetry between the cerebral hemispheres relates to emotions (15–17), the changes in the gamma band connects with happiness and sadness, and the reduction of alpha waves on different sides of the temporal lobe correlates with joy and sorrow (left side is sad, happy on the right) (18, 19).

Extracting emotion-related features to make larger the distance between classes and smaller the distance within classes is helpful to solving cross-database problems. Emotion-related EEG signal feature extraction methods include time domain [such as Hjorth extraction activity, mobility and complexity of EEG signals (20), higher-order crossover features used to describe the oscillation mode of a time series (21) and magnitude squared coherence estimate (22)], frequency domain [such as power spectral density features (23, 24)], time-frequency domain [such as time-frequency spectrum [TFS] features (25)], auto-regressive (26), asymmetric spatial pattern (27), entropy [such as differential entropy (7), sample entropy (28) and approximate entropy (29)], maximum relevance minimum redundancy method (30), common spatial patterns (31), filter bank common spatial pattern (32), higuchi fractal dimension (33), and so on. Regarding EEG feature types, all frequency bands or some frequency bands of delta, theta, alpha, beta, and gamma are mainly utilized (34). These features characterize the signal from different aspects, so a variety of effective features extracted from the signal can be better classified.

To train an excellent model, the user usually needs to collect enough marker data for calibration. This calibration process is typically time-consuming and laborious, which is a significant problem of practical use in emotional brain computer interface. Therefore, reducing or even eliminating the calibration process and realizing Plug-and-Play is a critical challenge for the brain–computer interface from the laboratory to real life. Transfer learning is a crucial technology that can solve this problem by using annotation data from other auxiliary users to help new users build models (35). However, due to individual differences, i.e., different users have different neural responses to the same event, such that need first to perform data distribution adaptation to alleviate the individual differences of EEG features. (36). To this end, in this paper we propose an unsupervised distributed adaptation method to align data between different users, that is, Euclidean alignment (EA) (37).

To improve EEG emotion recognition performance, performing deep neural networks to learn higher-level features would be useful to achieve good results, such as deep belief networks (7), recurrent neural networks (38), graph convolutional neural networks (39), transfer learning (40), and adversarial neural networks (41). Nevertheless, the recognition performance is limited to subject-dependent and cross-subject experiments under the same database, which is still far from realizing a practical emotional brain–computer interface. For this reason, we investigate an interesting and challenging problem in EEG emotion recognition, where training samples and test samples come from different emotional EEG databases. The preliminary research on EEG emotion recognition across data sets have demonstrated the significant drop of the recognition performance because of the inconsistency of feature distribution between the original training samples and test samples (42). Consequently, in this paper we will take advantages of the powerful high-level feature learning ability of deep learning technique to deal with the cross-database EEG emotion recognition problem.

The major contributions of this paper are summarized as follows:

(1) This paper proposes a novel recognition framework on the emotional EEG database, from raw data to recognition results, including preprocessing, feature engineering, classification recognition, and cross-database evaluation protocol.
(2) In feature engineering, we designed a time-frequency-spatial feature extraction method, combining forms of TFS, CNN, and bidirectional long and short memory network (BiLSTM) to extract multi-dimensional effectual features.
(3) Employing an unsupervised data alignment method to project data from different databases into the same space. While considering the inherent topological structure of the EEG electrodes, the preliminary TFS features are converted into three-dimensional tensors, which takes into account the information relationship between the electrodes.

This paper is organized as follows. Section 2 introduces emotion database, data processing methods, and experimental settings. Section 3 specifies the test results on the emotion database and the test results applied to the recognition of depression. Section 4 discusses the methods and results of this research. We conclude the paper in Section 5.

## 2. Materials and Methods

**Figure 1:**
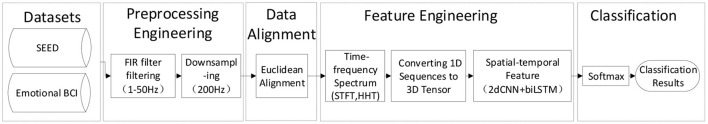
Cross-database emotion recognition framework based on electroencephalogram (EEG) signals. Emotional BCI Competition Database is the training set of the Emotional BCI in 2020WORLDROBOT COMPETITION-BCI CONTROL BRAIN ROBOT CONTEST Emotional BCI in 2020 WORLD ROBOT COMPETITION-BCI CONTROL BRAIN ROBOT CONTEST.

As shown in Figure 1, this section mainly introduces emotion database and the algorithms of preprocessing engineering and feature engineering, including filtering, downsampling, EA, short-time Fourier transform, Hilbert–Huang transform (HHT), conversion of 1D sequence to 3D tensor, and the spatiotemporal feature extraction model combined with convolutional neural network (CNN) and BiLSTM.

### 2.1. Emotion Database

One of the databases used in this study is SEED (43). This database includes 15 subjects, three sessions for each subject and three emotion categories under video stimulation (i.e., positive, neutral and negative). The data were downsampled to 200 Hz. A bandpass frequency filter from 0 to 75 Hz was applied. The data are cut into one sample per second, with a total of 152,730. The other database comes from the training set of the Emotional BCI in 2020 WORLD ROBOT COMPETITION—BCI CONTROL BRAIN ROBOT CONTEST Emotional BCI in 2020 WORLD ROBOT COMPETITION—BCI CONTROL BRAIN ROBOT CONTEST (Emotional BCI Competition Database), which includes 23 subjects, two sessions (from A and B, respectively), and three emotion categories under video stimulation (i.e., happy, sad, and neutral). The data samples rate of the Emotional BCI Competition Database is 100 Hz. The EEG signals are segmented in seconds and hence results in a total of 156,520 samples. Details of the two databases are shown in Table 1. It can be seen from the table that the two databases have differences in categories, subjects, sessions, and the number of samples. In the subsequent processing, the three categories of happy, sad, and neutral in the emotional BCI database correspond to the positive, negative, and neutral emotion, respectively.

**Table 1:** Details of the two experimental databases.

	**SEED**	**Emotional BCI Competition Database**
Category	Positive, neutral, negative	happy, sad, neutral
Channel	62	62
Subject	15	23
Session	3	2
Positive	49,680	49,110
Neutral	52,650	50,722
Negative	50,400	56,694
Sum	152,730	156,526

### 2.2. Preprocessing Engineering

EEG recordings measured by the scalp often contain noise and artifacts, such as blinking or movement, and cannot accurately represent signals from the brain. Therefore, it is necessary to preprocess the recorded EEG data. The preprocessing steps include converting or organizing the recorded EEG data, removing insufficient data, and segmenting the continuous original signal without changing the clean data. Appropriate band-pass filtering can effectively reduce the superimposed artifacts of various sources embedded in the EEG recording. Generally, the finite impulse response (FIR) filters are a good choice because they do not distort wave phases (44). EA maps each user's EEG signal to a new space so that the difference in the second-order statistics of the average covariance matrix of the mapped users is minimized, thereby implicitly reducing the difference in the original distribution. EA implements the above mapping for each user (auxiliary user and new user). Since different users have the same average covariance matrix after mapping, they tend to be more consistent in data distribution, meaning models trained on auxiliary users can be better applied to new users.

### 2.3. Data Alignment

EA is easy to perform and completely unsupervised, in which the basic idea of aligning EEG from different subjects (domains) is as follows (35): for all subjects, EA first calculates the arithmetic mean of all spatial covariance matrices.


(1) $$\begin{array}{r} \bar{R}=\frac{1}{N}\overset{N}{\underset{n=1}{\sum }}X_{n}{\left(X_{n}\right)}^{T} \end{array}$$

then performs the alignment by


(2) $$\begin{array}{r} {\tilde{X}}_{n}={\bar{R}}^{-\frac{1}{2}}X_{n} \end{array}$$

where $X_{n}\in {\mathbb{R}}^{c\times t}$ is the *n*th EEG trial, in which *c* is the number of EEG channels and *t* is the number of samples. The aligned EEG trials are whitened, and the average spatial covariance matrix of each subject is the identity matrix (45), so the EEG test distribution of different subjects is more consistent, which is meaningful for subsequent cross-database recognition.

### 2.4. Time-Fequency Spectrum

The EEG signal is non-linear and non-stationary, so its statistical properties (for example, spectral density) will change greatly over time. Spectrum estimation cannot identify its time-varying spectral components and cannot perform time-frequency positioning simultaneously. Time-frequency analysis technology is capable of revealing the time-varying frequency spectrum of non-stationary EEG signals and can provide a joint time-frequency distribution (TFD) of signal power (46). This paper adopts two methods of short-time Fourier transform (STFT) (47–49) and HHT (50, 51) for time-frequency spectrum (TFS) analysis.The method of calculating TFS using STFT and HHT comes from Song et al. (25).

STFT spectrum is calculated by


(3) $$TFS_{STFT}(t,f)\;=\;|\int_{-\infty }^{+\infty }w(\tau -t)x(\tau )e^{-j2\pi f\text{τ}}\text{d}\tau {|}^{2}$$

where *x*(*t*) is the time series and *w*(τ−*t*) is the short-time analysis window.

The Hilbert–Huang spectrum is calculated based on HHT. HHT processing non-stationary signals include three basic processes. First, the empirical mode decomposition (EMD) method is used to decompose a given signal into a number of intrinsic mode functions (IMF),


(4) $$\begin{array}{r} x\left(t\right)=\overset{K}{\underset{i=1}{\sum }}IMF_{i}\left(t\right)+r_{K}\left(t\right) \end{array}$$

where *r*_*K*_(*t*) represents the residual of a constant or monotonic signal. These IMFs are components that meet certain conditions. Then, perform Hilbert transform on each IMF to obtain the corresponding Hilbert spectrum, that is, represent each IMF in the joint time-frequency domain. An analytic signal reconstructed by a conjugate pair (*IMF* and $IMF_{k}^{*}$) can be formulated as


(5) $$\begin{array}{r} Z_{k}=IMF_{k}\left(t\right)+jIMF_{k}^{*}=A_{k}\left(t\right)e^{j\theta_{k}\left(t\right)} \end{array}$$

where *A*_*k*_(*t*) represents the instantaneous amplitude of *Z*_*k*_(*t*) and θ_*k*_(*t*) denotes the instantaneous phase of IMFk(t). Finally, summarizing all Hilbert spectra of IMF will get the Hilbert spectra of the original signal. The original time series x(t) can be obtained by


(6) $$\begin{array}{r} x\left(t\right)=\overset{K}{\underset{i=1}{\sum }}A_{k}\left(t\right)e^{j2\pi \int f_{k}\left(t\right)\text{d}t} \end{array}$$

and the instantaneous frequency can be evaluated by


(7) $$\begin{array}{r} f_{i}\left(t\right)=\frac{1}{2\pi }\frac{d\theta_{i}}{dt} \end{array}$$

where the squared amplitude $A_{k}^{2}\left(t\right)$ and instantaneous frequency *f*_*k*_(*t*) form the time-frequency spectrum.

### 2.5. Convert 1D Feature Sequence to 3D Tensor

Due to a large amount of noise in the EEG signal and the difficulty in capturing the unobvious relationship between the EEG signal and certain brain activities, the practical interpretation of the EEG signal is still challenging. Most of the existing studies only treat EEG as a chain sequence, ignoring the complex dependence between adjacent signals or the need to convert EEG, such as converting EEG waves into images (52).

**Figure 2:**
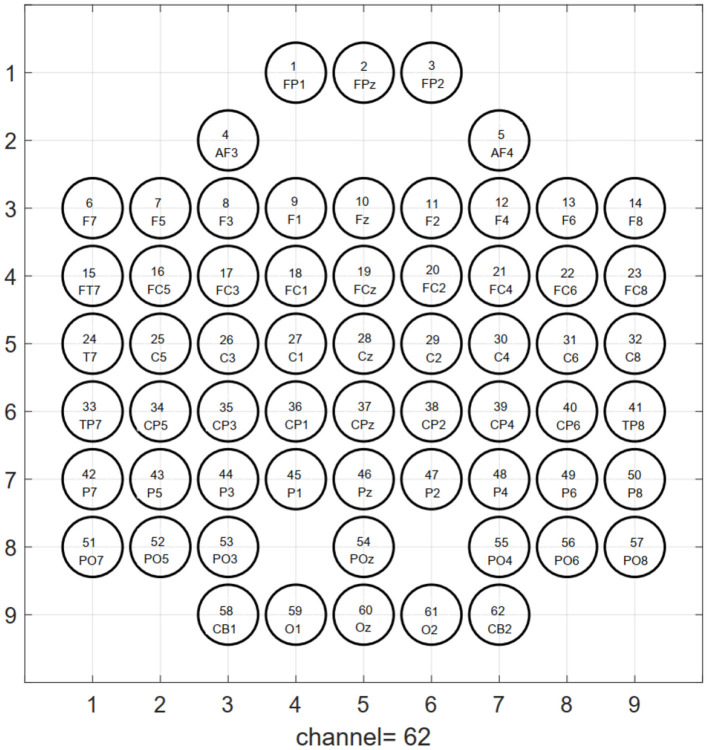
Topological structure map of 64-electrode channels mapped to a two-dimensional picture. The circle represents the electrode, and the label inside is the serial number and name of the electrode. The left and right mastoid electrodes (M1, M2) of the 64-lead electrodes are reference electrodes when collecting signals, so they are not used as signal input for emotion recognition.

According to the inherent topological structure of the EEG channel, as illustrated in Figure 2, the one-dimensional sequence data $S_{t}=\left[s_{f}^{1},...,s_{f}^{c},...,s_{f}^{C}\right]$ (where $s_{f}^{c}$ is the TFS feature of the *c*th electrode channel at frequency *f*) after extracting the TFS feature is mapped into a three-dimensional tensor $T_{n}\in R^{H\times W\times F}$, where the first dimension *H* is height, the second dimension *W* is width, and the third dimension *F* is channel (i.e., the number of features extracted per channel) of the *n*th EEG trial. The conversion function of 1D feature sequence to 3D tensor *T*_*n*_(*H, W, f*) is,


(8) $$\begin{array}{r} T_{n}\left(H,W,f\right)=\left[\begin{array}{ccccccccc} 0 & 0 & 0 & s_{f}^{1} & s_{f}^{2} & s_{f}^{3} & 0 & 0 & 0\\ 0 & 0 & s_{f}^{4} & 0 & 0 & 0 & s_{f}^{5} & 0 & 0\\ s_{f}^{6} & s_{f}^{7} & s_{f}^{8} & s_{f}^{9} & s_{f}^{10} & s_{f}^{11} & s_{f}^{12} & s_{f}^{13} & s_{f}^{14}\\ s_{f}^{15} & s_{f}^{16} & s_{f}^{17} & s_{f}^{18} & s_{f}^{19} & s_{f}^{20} & s_{f}^{21} & s_{f}^{22} & s_{f}^{23}\\ s_{f}^{24} & s_{f}^{25} & s_{f}^{26} & s_{f}^{27} & s_{f}^{28} & s_{f}^{29} & s_{f}^{30} & s_{f}^{31} & s_{f}^{32}\\ s_{f}^{33} & s_{f}^{34} & s_{f}^{35} & s_{f}^{36} & s_{f}^{37} & s_{f}^{38} & s_{f}^{39} & s_{f}^{40} & s_{f}^{41}\\ s_{f}^{42} & s_{f}^{43} & s_{f}^{44} & s_{f}^{45} & s_{f}^{46} & s_{f}^{47} & s_{f}^{48} & s_{f}^{49} & s_{f}^{50}\\ s_{f}^{51} & s_{f}^{52} & s_{f}^{53} & 0 & s_{f}^{54} & 0 & s_{f}^{55} & s_{f}^{56} & s_{f}^{57}\\ 0 & 0 & s_{f}^{58} & s_{f}^{59} & s_{f}^{60} & s_{f}^{61} & s_{f}^{62} & 0 & 0 \end{array}\right] \end{array}$$

which is the **f**th channel features. Among them, the positions without electrodes were filled with zeros. Each generated data grid contains spatial information of brain activity.

### 2.6. 2dCNN+BiLSTM

**Figure 3:**
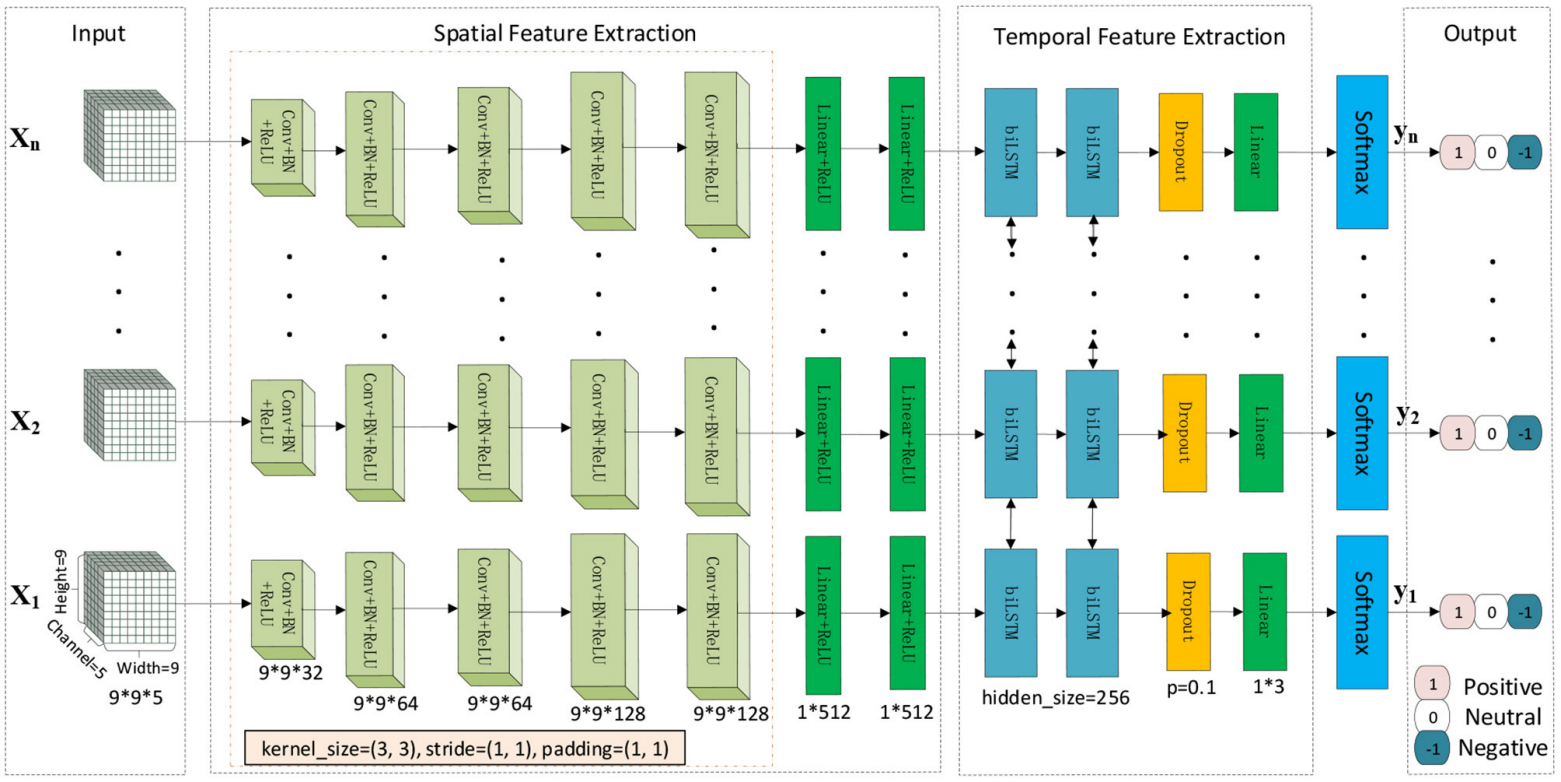
Cascade 2dCNN+BiLSTM architecture.

We designed a cascaded deep convolutional recurrent neural network framework, as shown in Figure 3, to capture the spatiotemporal features of EEG. The model's input is the converted 3D tensor *T*_*n*_ that a 3D data structure containing space and time information. First, 2D CNN extracts the spatial features of each data, BiLSTM extracts temporal features, a fully connected layer receives the output of the last step of BiLSTM, and then uses the softmax layer for final emotion prediction.

This study constructed a 2dCNN+BiLSTM model to learn a good spatiotemporal representation for multi-channel EEG. The diagram for this deep spatiotemporal network is illustrated in Figure 3. Since each EEG segment with the duration of 1 s is treated as one sample, we conduct time-frequency spectrum feature (STFT and HHT) extraction for each sample, which was fed into the deep network for deep feature extraction. Each 1-s sample is denoted by *X*_*i*_(*i* = 1, 2, …, *n*) and treated as a spatial image with five channels. Five convolutional layers were followed by ReLU to learn non-linear local spatial features, in which a 3 x 3 convolutional kernel was used. Following the convolutional layers, the fully connected layers were utilized to learn global spatial features. Existing studies showed that spatial features for a temporal signal are insufficient for discriminant information representation. We also employed the BiLSTM to learn temporal representation.

### 2.7. Experimental Settings

First, we utilize the FIR filter to perform 50-order 1–50 Hz band-pass filtering on the EEG original signal, downsampling on the Emotional BCI data to 200 Hz to be consistent with the SEED data, and then perform EA. Then extract the relative energy of the five frequency bands for each electrode channel [i.e. delta (1–4 Hz), theta (4–8 Hz), alpha (8–14 Hz), beta (14–30 Hz), and gamma (30–50 Hz)] using STFT and HHS, respectively. The number of features extracted from each sample is 5*62 = 310, then converted into a 3D tensor of 9*9*5. Then feed the 3D tensor to spatiotemporal network for training, the batch size is 32, the frame length is 12 (i.e., 12 s), the epoch set to 100, the cross-entropy used as loss function, the optimizer selects SGD, the learning rate initialized to 0.005. The update calculation is *lr* = *init*_lr*(0.95^*epoch*//10^), where *init*_*lr* is the initial learning rate.

## 3. Results

### 3.1. Emotion Recognition Results

In order to test the performance of the emotion recognition framework system built in various aspects, three protocols are proposed. In the three protocols proposed in this study, the training set and test set data are completely non-overlapping, and the test set data and labels are not used in the training process. The training set and test set of the first two protocols are from different databases. The third protocol is the leave-one-subject-out method. Considering the imbalance of the category, in addition to calculating the accuracy, the F1 score is also calculated. We applied this model to depression recognition and performed five-fold cross-validation.

**Figure 4:**
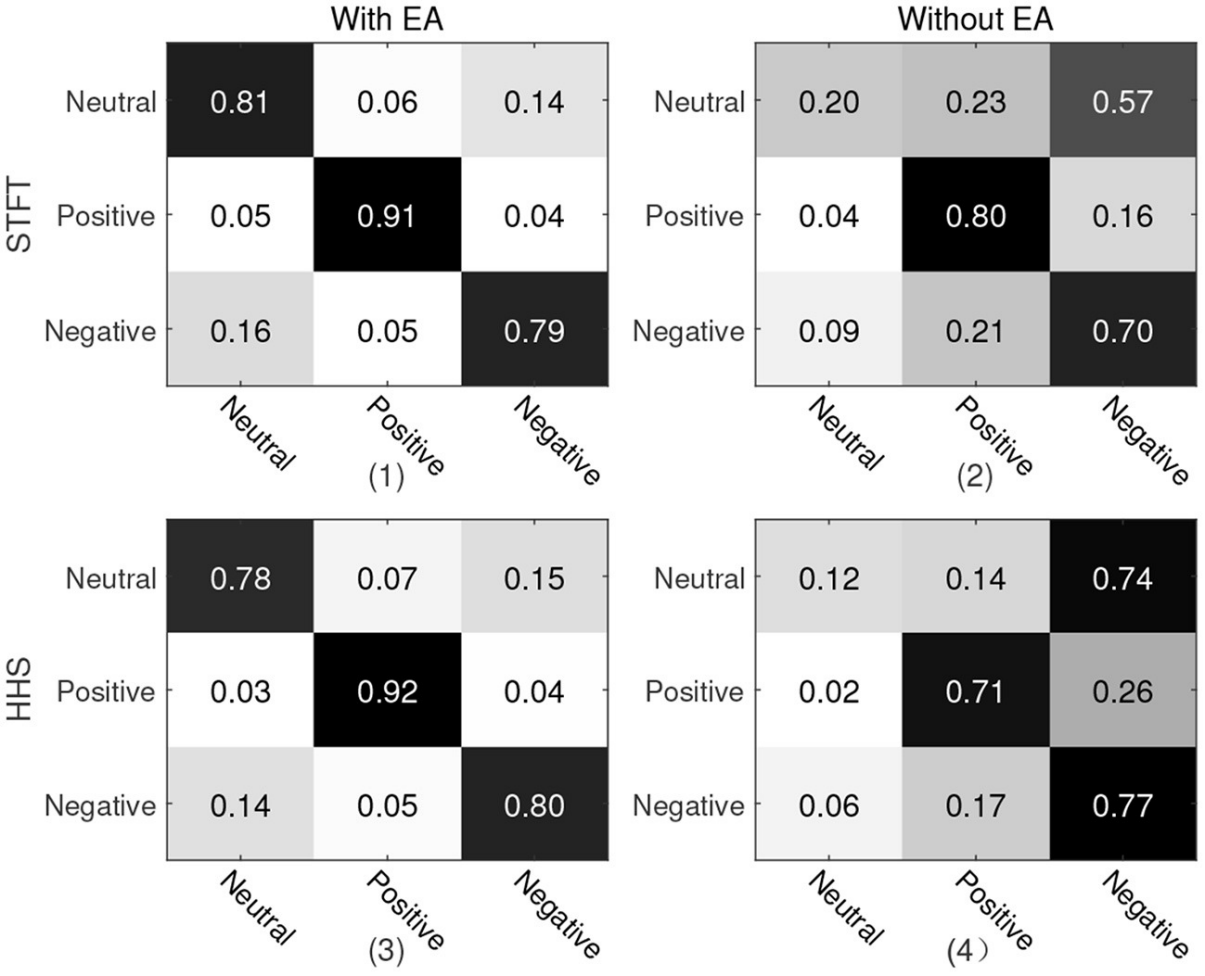
Confusion matrix of protocol 1. The vertical axis is the true label and the horizontal axis is the predicted label. (1) and (2) are the recognition results of STFT features; (3) and (4) are the recognition results of HHS features; (1) and (3) are with Euclidean alignment (EA) module, while (2) and (4) are the recognition result without the EA module.

For the first protocol, all data of Emotional BCI competition database are used as the training set, and all data of SEED are used as the test set. For two different manual features, the recognition accuracy and weighted average F1-score are shown in Table 2. It can be seen from the table that the manual feature recognition effect extracted by the STFT method is better. In order to show the true prediction of each category, the confusion matrix of the classification accuracy is analyzed. we present a confusion matrix exploiting the features of STFT and HHS shown in Figure 4, from which we can see that neutral emotion has the highest recognition rate among the three types of emotions, whether it is STFT or HHS features. The recognition rate of the three types of emotions under the STFT feature is higher than that of the HHS feature.

**Table 2:** Recognition results on emotional database.

**No**.	**Protocol**	**STFT**		**HHS**	
	**Training set → Test set**	**Acc(%)**	**F1**	**Acc(%)**	**F1**
1	Emotional BCI Competition Database → SEED	83.56	0.84	83.60	0.84
2	SEED → Emotional BCI Competition Database	74.33	0.72	70.26	0.70
3	Leave-One-Subject-Out	81.58	0.80	79.29	0.77

**Figure 5:**
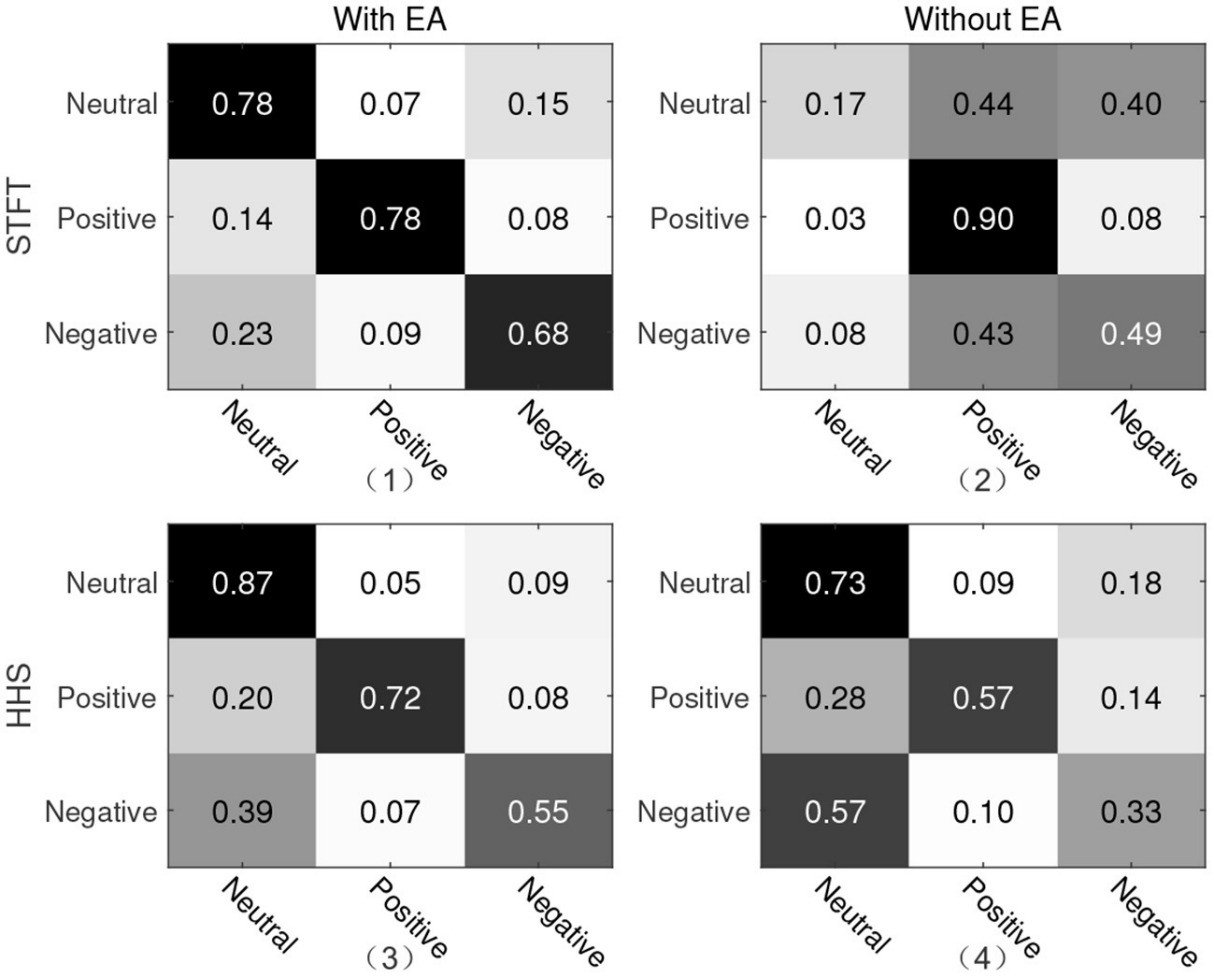
Confusion matrix of protocol two. The vertical axis is the true label and the horizontal axis is the predicted label. (1) and (2) are the recognition results of STFT features; (3) and (4) are the recognition results of HHS features; (1) and (3) are with Euclidean alignment (EA) module, while (2) and (4) are the recognition result without the EA module.

For the second protocol, all data of SEED are used as the training set, and all data of Emotional BCI Competition Database are used as the test set. For two different manual features, the recognition accuracy and weighted average F1-score are shown in Table 2. The recognition rate under the STFT feature is 4.07% higher than that of the HHS feature, but it is about 9% lower than the protocol 1. Similarly, we present a confusion matrix using the features of STFT and HHS shown in Figure 5. The recognition rate of the three categories under the STFT feature is relatively balanced, while the recognition rate of positive emotion under the HHS feature is significantly higher than the other two categories.

For the third protocol, the recognition results of two databases are shown in Table 2 including the accuracy and weighted average F1-score where the recognition results are sorted according to the database (i.e., the recognition results of the subjects in each database are averaged) and the average recognition rate of all subjects is calculated. It can be seen from the table that the recognition result under the STFT feature is slightly higher than HHS.

**Figure 6:**
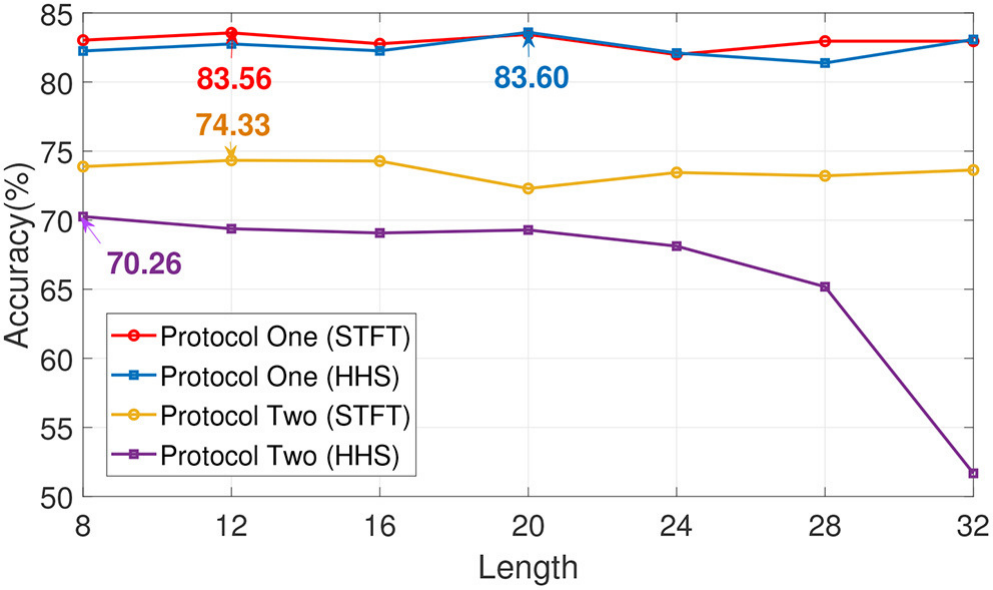
The impact of time length selection on recognition rate. The length of time is in seconds.

In order to explore the influence of EA on experiment, an ablation experiment was performed on this module. As shown in Figures 4, 5 and Table 3, the difference between the recognition results of the EA module and the absence of the EA module is very obvious, whether it is protocol 1 or 2. At the same time, in order to explore the timing relationship between EEG emotional frames, the frame length is selected from 8 to 32, and the step size is 4 during training. Experiments were carried out on protocols one and two, and the experimental results are shown in Figure 6.

**Table 3:** EA ablation experiment results.

**Training set → Test set**	**TFS**	**EA**	**Acc(%)**	**F1**
Emotional BCI Competition	STFT	With EA	**83.56**	**0.84**
Database → SEED				
		Without EA	57.29	0.54
	HHS	With EA	**83.60**	**0.84**
		Without EA	53.84	0.49
SEED → Emotional	STFT	With EA	**74.33**	**0.72**
BCI Competition Database				
		Without EA	52.40	0.48
	HHS	With EA	**70.26**	**0.70**
		Without EA	53.46	0.53

*Bold value indicate the same experimental conditions, the maximum index with or without EA comparison*.

### 3.2. Depression Recognition Results

**Figure 7:**
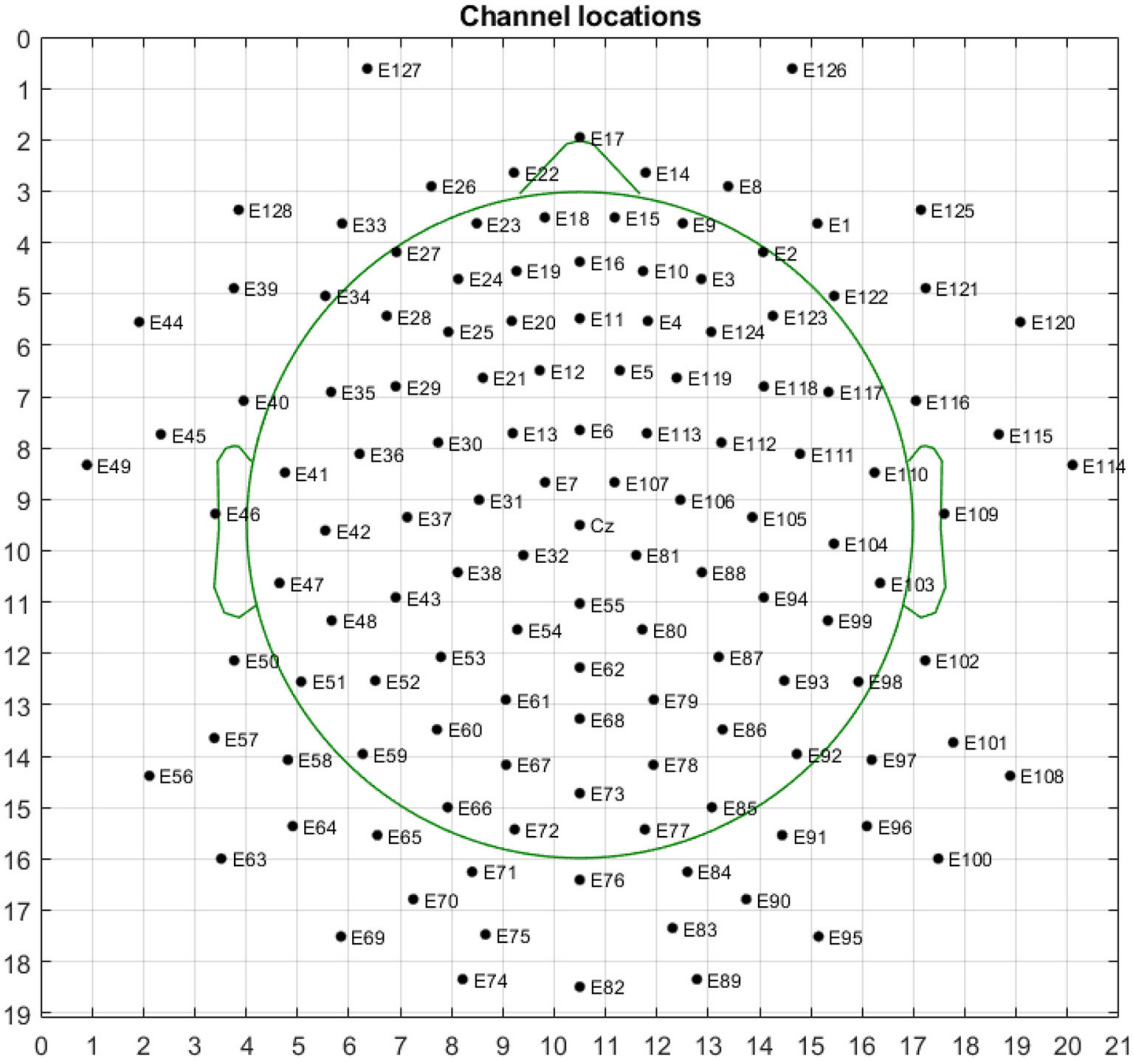
Topological structure map of 128-electrode channels mapped to a two-dimensional picture. The circle represents the electrode, and the label inside is the serial number and name of the electrode.

**Figure 8:**
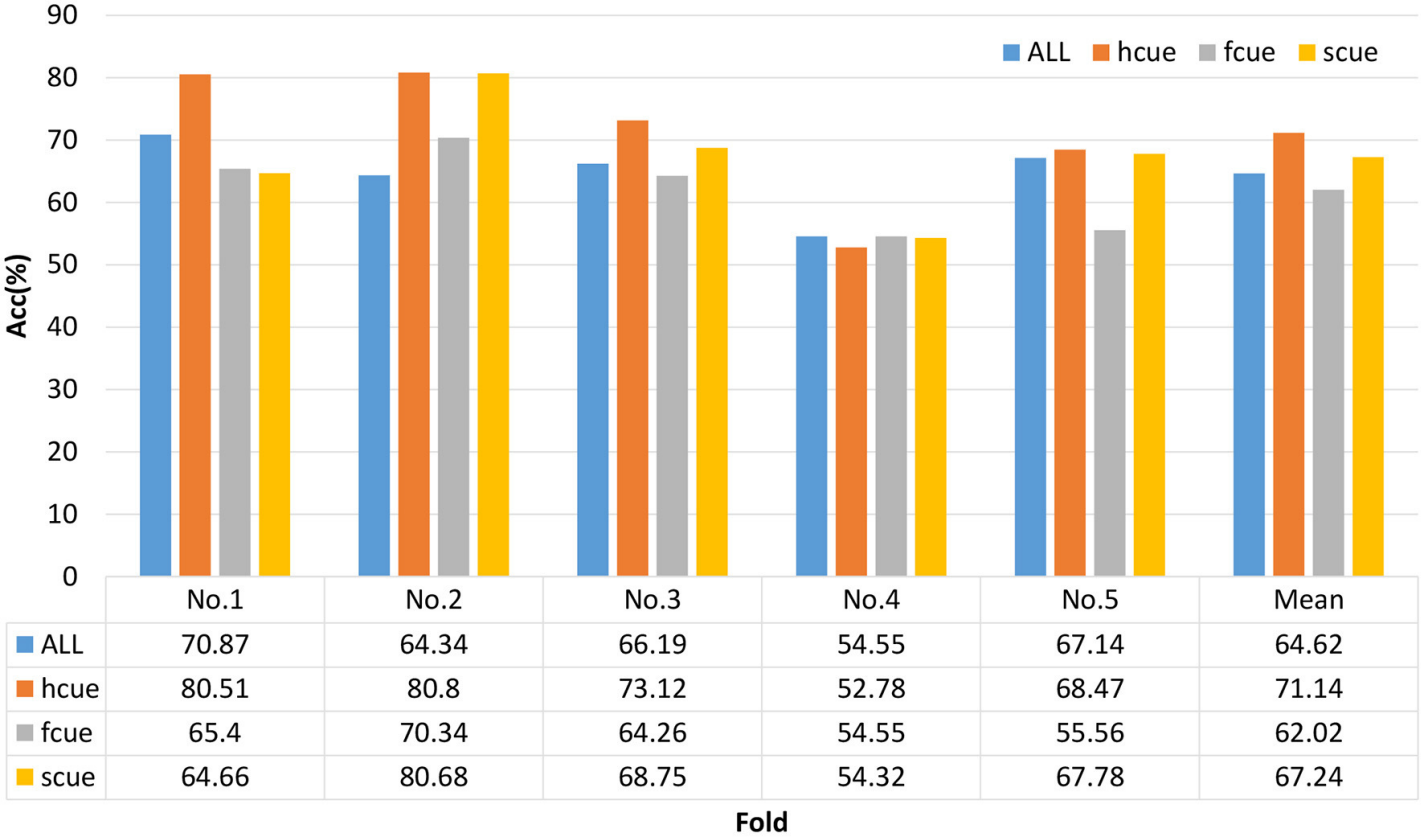
Recognition results of spatiotemporal neural network on depression database.

We chose a multi-modal open dataset for depression recognition, i.e., the MODMA dataset. The dataset includes 128-channel event-related potential recordings, of which 24 major depressive disorder subjects and 29 healthy controls, the age range is 16–52 years old (53–55). Since the number of electrodes in the database is 128 and the topology is shown in Figure 7, the size of the three-dimensional matrix mapped to it is 21*19*5. Note that 53 subjects, including 24 outpatients and 29 healthy controls, were divided into fivefold. Due to uneven data, the first three folds made up of 5 depressed and 6 normal subjects of each one, and the last fold included 4 depressed and 5 normal subjects. The recognition result of each fold is shown in Figure 8. The ERP experiment is a dot-probe task, and its cue stimuli include three kinds of emotional-neutral face pairs, namely Happy-Neutral (“hcue”), Fear-Neutral (“fcue”), and Sad-Neutral (“scue”). Therefore, we not only tested all the experiments but also identified depressed patients and normal subjects on different stimuli. Among them, the overall recognition rate on “hcue” is the highest, reaching 71.14%.

## 4. Discussion

This paper proposes a complete pipeline from preprocessing to EEG-based emotion recognition, with a recognition rate of over 80%. The preprocessing part follows with the unsupervised EA method to map the data of different databases to the same space, where STFT, CNN, and BiLSTM are combined to extract multi-domain features in the time-frequency space. Before the CNN operation, according to the spatial arrangement of the EEG electrodes, the one-dimensional time series feature is converted into a three-dimensional tensor, such that the correlation between EEG electrodes can be fully considered. Moreover, we use 2D CNN to extract spatial features, and BiLSTM to capture the timing relationship of features.

It can be seen from the confusion matrix of protocol 1 that the recognition rates of the three categories under the two methods of STFT and HHS are relatively balanced, and the positive emotion recognition rate is the highest. The neutral emotion recognition rate under the HHS method of protocol 2 is the highest, and the negative emotion recognition rate is the lowest. There is a 9% difference between the accuracy of protocols 1 and 2. Since the three categories of data in the Emotional BCI database are more diversified (the first 15 people and the bottom eight people in the three categories of the Emotional BCI database in Table 1 are different).

**Figure 9:**
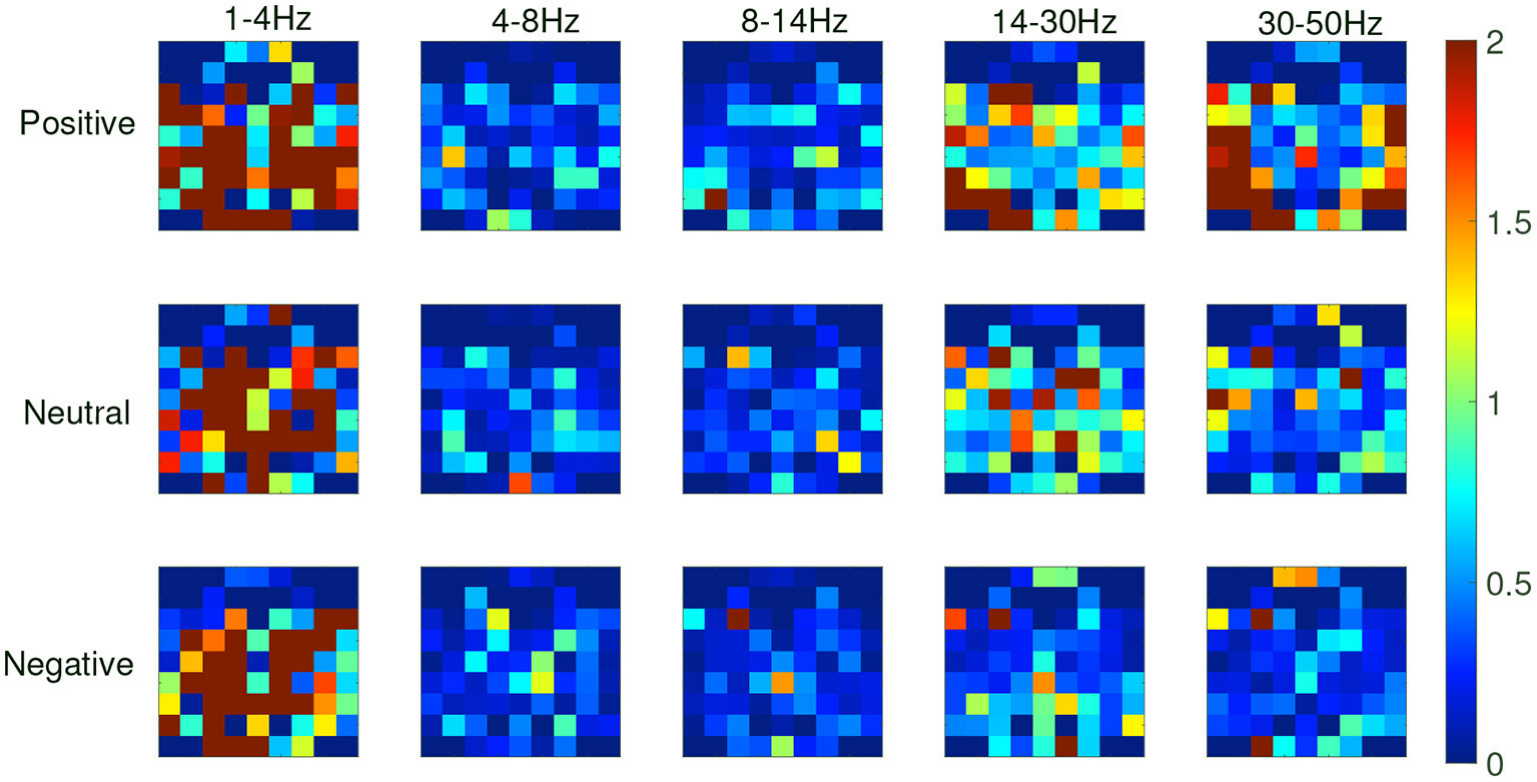
The time-frequency spectrum (TFS) characteristic relative energy map (based on the short-time Fourier transform [STFT] algorithm) corresponds to the electrode arrangement in Figure 2.

**Figure 10:**
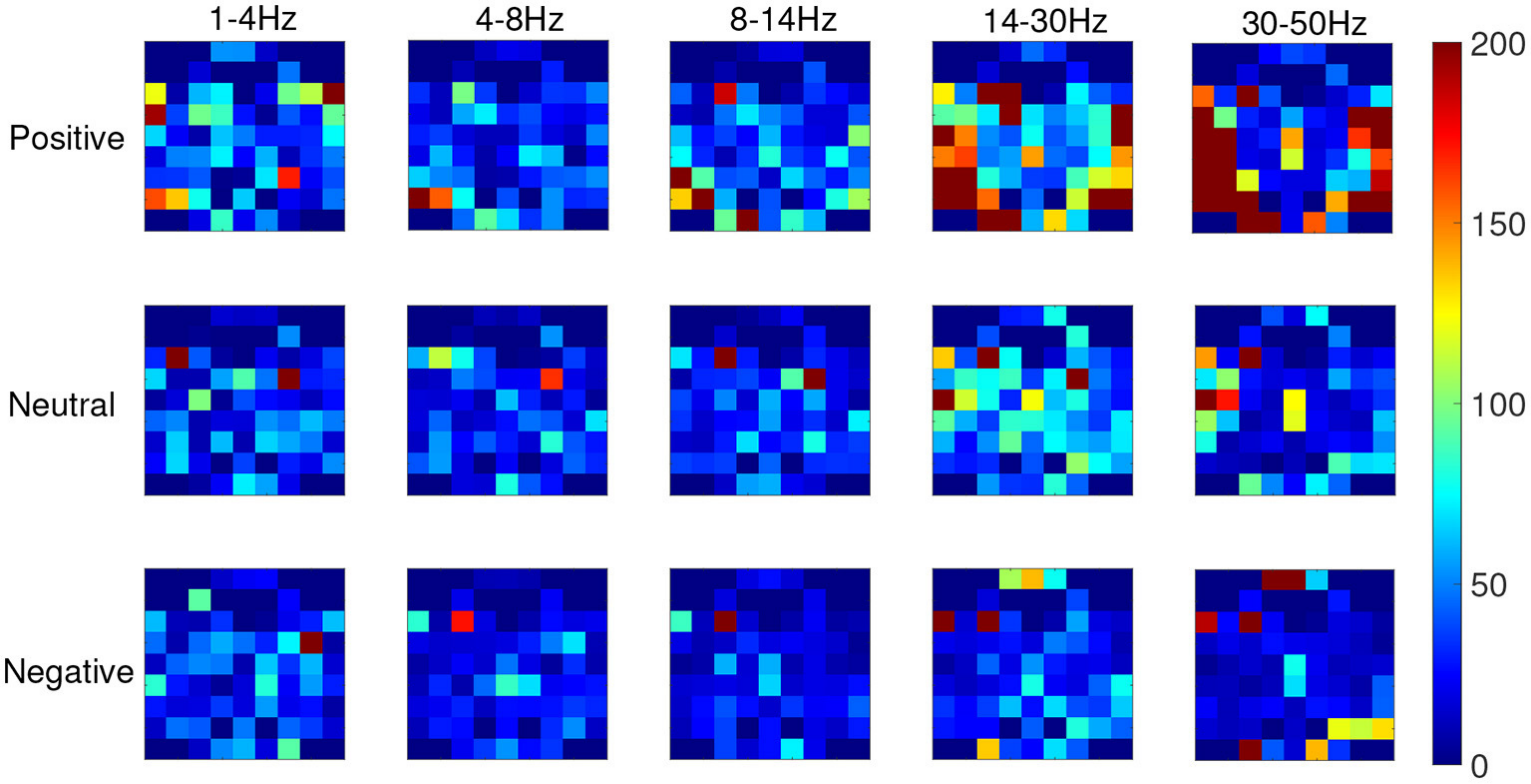
The time-frequency spectrum (TFS) characteristic relative energy map (based on the HHS algorithm) corresponds to the electrode arrangement in Figure 2.

From the recognition results of all protocols, the accuracy and F1 score of the TFS features extracted by STFT are higher than those of the HHS method. Figure 9 shows the STFT method and Figure 10 shows the HHS method. The three categories are displayed in five frequency bands, and each spectrum is shown per the electrode arrangement in Figure 2. The features extracted by the two methods are pretty different in the high-frequency range. The relative energy of the two frequency bands, beta (14–30 Hz) and gamma (30–50 Hz), under the STFT method, is relatively high, and the three categories have apparent differences. In contrast, the HHS method has relatively high positive and neutral relative energies in these two frequency bands. Negative emotions have always been low energy in the entire frequency band. Hence, the recognition rate of the HHS method is lower than that of STFT, and it performs well in positive and negative emotions.

To further validate the proposed method, we compared our model with the start-of-the-art methods. Table 4 presents a summary of the current subject-independent recognition algorithms on the SEED database, including linear support vector machine (SVM) (56), kernel principal component analysis (KPCA) (57), transfer component analysis (TCA) (58), transductive parameter transfer (TPT) (59), domain adversarial neural network (DANN) (60), dynamical graph convolutional neural network (DGCNN) (39), bi-hemispheres domain adversarial neural network (BiDANN) (61), BiDANN-S (41), hierarchical spatial-temporal neural network (R2G-STNN) (62), and instance-adaptive graph (IAG) (63). It can be seen from the table that our method has achieved the highest accuracy and the smallest standard deviation. Unlike these methods, our training set adds the Emotional BCI database. The training set's increase makes the training model's generalization better, proving that the proposed method can effectively extract spatiotemporal multi-view features and classify emotions well across databases or subjects.

**Table 4:** The mean accuracies (Acc) and standard deviations (Std) on SEED dataset for subject-independent EEG emotion recognition experiment.

**Method**	**Acc/Std(%)**
SVM (56)	56.73/16.29
KPCA (57)	61.28/14.62
TCA (58)	63.64/14.88
TPT (59)	76.31/15.89
DANN (60)	75.08/11.18
DGCNN (39)	79.95/09.02
BiDANN (61)	83.28/09.60
BiDANN-S (41)	84.14/06.87
R2G-STNN (62)	84.16/07.63
IAG (63)	86.30/06.91
ours	**86.42/05.26**

## 5. Conclusions

This study designed a complete pipeline from preprocessing to the classification of emotion recognition based on EEG, which achieved a correct rate of more than 80%. It is significant that we apply this model to the recognition of depression based on EEG signals. The preprocessing combined with the unsupervised EA method maps the data of different databases to the same space. The three methods of STFT, CNN, and BiLSTM are combined to extract the time-frequency-space multi-domain features. Before the CNN operation, the one-dimensional time series feature was converted into a three-dimensional tensor according to the spatial arrangement of the EEG electrodes. In the future, we will study end-to-end real-time emotional brain–computer interfaces for depression recognition.

## Data Availability Statement

Publicly available datasets were analyzed in this study. This data can be found here: https://bcmi.sjtu.edu.cn/~seed/index.html; http://modma.lzu.edu.cn/data/application/#data_1; https://oneuro.cn/n/index.html?code=d349d58c825b4041a0e53ea55b5157ae/state=#/chinabci, Emotional BCI.

## Author Contributions

HC and YZ: conceptualization. HC: methodology and writing and original draft preparation. WZ: formal analysis and funding acquisition. CT: investigation. JZ: resources. XL: data curation. CT and JZ: review and editing. All authors have read and agreed to the published version of the manuscript.

## Funding

This work was supported in part by the National Natural Science Foundation of China under grant 62076064, 61921004, 62076195, and 81971282, and the Zhishan Young Scholarship of Southeast University.

## Conflict of Interest

The authors declare that the research was conducted in the absence of any commercial or financial relationships that could be construed as a potential conflict of interest.

## Publisher's Note

All claims expressed in this article are solely those of the authors and do not necessarily represent those of their affiliated organizations, or those of the publisher, the editors and the reviewers. Any product that may be evaluated in this article, or claim that may be made by its manufacturer, is not guaranteed or endorsed by the publisher.
